# Effect of Heat Input on the Microstructure and Mechanical Properties of Local Dry Underwater Welded Duplex Stainless Steel

**DOI:** 10.3390/ma16062289

**Published:** 2023-03-13

**Authors:** Yu Hu, Yonghua Shi, Kai Wang, Jiqiang Huang

**Affiliations:** 1 School of Mechatronic Engineering and Automation, Foshan University, Foshan 528231, China; huyuscnu@163.com (Y.H.); hfutwk927@fosu.edu.cn (K.W.); 2 School of Mechanical and Automotive Engineering, South China University of Technology, Guangzhou 510640, China; 3 School of Mechanical Engineering, Beijing Institute of Petrochemical Technology, Beijing 102627, China; huangjiqiang@bipt.edu.cn

**Keywords:** local dry underwater welding, duplex stainless steel, flux-cored arc welding, microstructure, mechanical performance

## Abstract

Duplex stainless steel welded metals were underwater local dry prepared on S32101 lean duplex stainless steel trapezoidal groove plates with a self-made drain cover employing Supercore 2205P flux-cored filler wire. Different heat inputs were employed to investigate the effects on mechanical characteristics and the microstructure of welded metals. The results demonstrated that as the heat was applied, austenite concentrations in the weld metals increased. It was found that the austenite concentration and the fraction of Σ3-austenite twin-grain boundaries followed the same trends. With increasing heat input, the recrystallized ferrite and austenite grains initially decreased and subsequently increased, whereas the fraction of interphase boundaries between special ferrite and austenite exhibited the reverse trend. With a heat input of 1.4 kJ/mm, the toughness and plasticity of the weld metals were enhanced by an increase in austenite content, Σ3 recrystallized grains, and austenite twin-grain boundaries. The plasticity and tensile strength values of the welded metal changed more when the heat input was raised from 1.0 to 1.2 kJ/mm than when it was raised from 1.2 to 1.4 kJ/mm. Considering energy conservation, it is recommended to adopt 1.2 kJ/mm for welding heat input.

## 1. Introduction

The flourishing growth of offshore, nuclear power, and shipbuilding has increased the use of underwater welding technology [1]. Local dry underwater welding has proven to be a potential joining method for the construction and repair of engineering structures under water [2]. The welding process was conducted underwater, and the water around the welding zone was drained through a drain cover in local dry underwater welding [3]. Different from wet underwater welds, which are always accompanied by defects such as pores and cracking, local dry underwater welding quality is almost comparable to dry underwater welding, but with a lower equipment cost [4,5].

High strength and good corrosion resistance are in harmony when combined skillfully to create ferrite–austenite duplex stainless steel [6,7]. Ocean petroleum platforms, nuclear power plants, pipelines of gas and oil, and other offshore and marine engineering projects are all being constructed with duplex stainless steel [8]. For the construction and maintenance of underwater duplex stainless steel structures, it is essential to investigate the underwater welding duplex stainless steel. Duplex stainless steel has good weldability. Almost all welding methods can be used to weld duplex stainless steel. The American Welding Society (AWS) has acknowledged over 90 different forms of welding techniques, such as metal arc welding (GMAW), gas tungsten arc welding (GTAW), flux-cored arc welding (FCAW), shielding metal arc welding (SMAW), and regulated metal deposition (RMD) in a chronological order. In 2004, Miller Electric Mfg. presented a new welding method named RMD by modifying the existing short-circuiting GMAW process. The RMD method is mostly used for pipeline root-pass welding, due to that it can maintain a consistent arc length independent of wire stick-out and yield spatter-free weld. Studies have been made on the surface morphology, microstructure, and mechanical performance of RMD welding of the ASTM A387-11–2 Steel Plates [9,10]. In this study, considering the project requirements and the existing experimental conditions, we use FCAW as the welding method for the underwater local dry welding of duplex stainless steel. In local dry underwater FCAW, in addition to the drainage, slag formed during welding with flux-cored wire further protected the weld pool from the surrounding water environment.

Some researchers have made a series of explorations on the underwater welding of duplex stainless steel. Underwater wet welding of 2205 duplex stainless steels with no cracks or slag inclusions was achieved using Ni-based self-shielded flux-cored wire and coated electrode [11,12]. The good weldability of local dry underwater welding of 2205 duplex stainless steel with gas metal arc welding at a water depth of 0.5 m was confirmed [13]. Further, local dry underwater welded duplex stainless steel was studied in terms of microstructure evolution, weldability, and mechanical properties at varying simulated water depths utilizing FCAW [14]. With the aid of drainage, they discovered that the weld formability is good at various water depths; in addition, the water depth has a significant impact on the austenite content, proportion of Kurdjumov-Sachs ferrite–austenite interphase boundaries, impact toughness, and microhardness.

The comprehensive performance of duplex stainless steel can be attributed to its almost equal fractions of austenite and ferrite phases in the microstructure [15]. The duplex stainless steel welded metal’s microstructure is affected largely by the welding heat input [16]. Higher heat input results in slower cooling rates, longer times for austenite to transform from ferrite, and greater austenite concentration in the welded metal [17]. Comparing the cooling rates of air welding and wet underwater welding, local dry underwater welding shows no significant differences. This study examines the impact of heat input on the mechanical characteristics and microstructure of local dry underwater welded duplex stainless steel utilizing flux-cored wire. During the cooling process, the correlation between microstructure development, heat input, and mechanical characteristics of welded metals was examined. The results will be utilized as a guide for developing a local dry underwater welding procedure for duplex stainless steel.

## 2. Materials and Methods

Lean duplex stainless steel UNS S32101 was used as the base metal (BM) in this study with dimensions of 300 mm × 100 mm × 10 mm. Welding was performed using Lincoln Supercore 2205P 1.2 mm flux-cored wire. The chemical compositions of the filler material and steel are displayed in Table 1.

**Figure 1:**
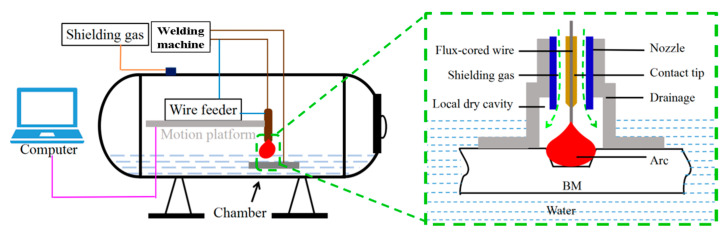
Schematic illustration of local dry underwater FCAW.

**Figure 2:**
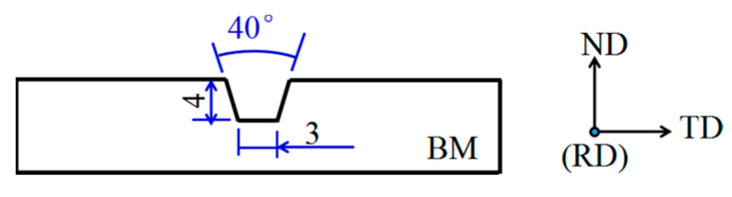
The designed groove for welding.

The test equipment includes a water storage room, power source for FCAW, and platform for three-dimensional motion, including drainage and other ancillary facilities, as illustrated in Figure 1. Before welding, the chamber was filled with fresh tap water until its surface was 10 mm above the BM sample’s surface. As illustrated in Figure 2, the base metal sheet has a trapezoidal groove. One weld pass was deposited to fill the groove. Table 2 lists the welding process parameters: 200 A current; 30 V arc voltage; 18 mm electrode extension; three welding speeds of 5.6, 4.8, and 4 mm/s; and three heat inputs of 1.0, 1.2, and 1.4 kJ/mm. Protection was performed at 45 L/min utilizing 99.999% CO_2_ as the shielding gas. The drainage and shielding gas together form a waterless arc combustion chamber.

**Figure 3:**
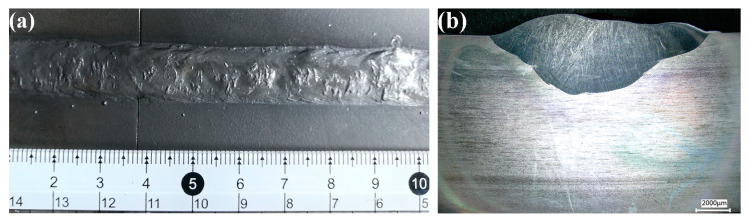
The appearance and cross-section images of the weld obtained with 1.2 kJ/mm (**a**) appearance image and (**b**) cross-section image.

All welds have little of obvious surface defects except for a little splash. Similar welds were also obtained under different simulated water depths [14]. All welds are uniform in shape, and the transition between weld metal and base metal is smooth. In this paper, the welding process is carried out in the trapezoidal groove as shown in Figure 2, and these parameters can both fill the trapezoidal groove. The welding heat input was changed by changing the welding speed, so that the weld with larger heat input has more deposited metal and the weld is relatively more plump. The appearance and cross-section images of the weld obtained with 1.2 kJ/mm are shown in Figure 3.

**Figure 4:**
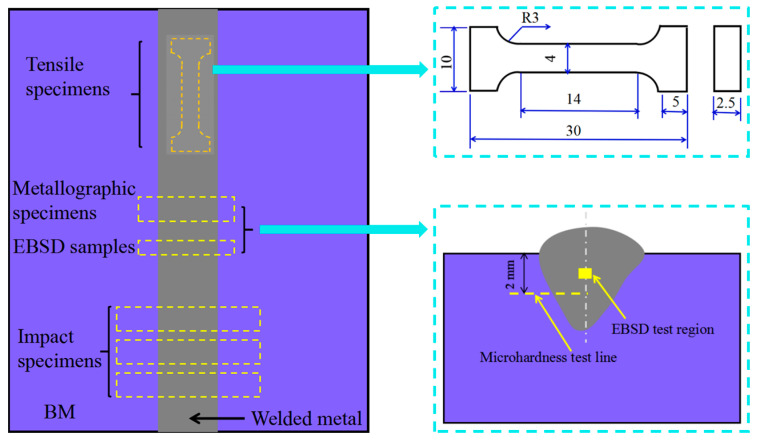
Sampling locations and sizes of the relevant tested specimens.

Figure 4 depicts the sampling locations for the Charpy V-notch impact specimens, tensile specimens, and metallographic specimens. Metallographic samples were polished with a diamond suspension to expose the weld cross-section; and etched with 30 mL HCl, 1 g K_2_S_2_O_5_, and 60 mL distilled water for 10–15 s. Phase fraction calculation, interface boundary evaluation, and grain size calculation of the welded metal were performed on the transverse direction-rolling direction plane by an electron backscatter diffraction (EBSD) analyzer. The normal direction was parallel to the welding direction. The welds were put through tensile tests using an electronic universal material testing equipment operated by a microprocessor at a loading rate of 0.5 mm/min. Charpy V-notch impact tests on the welded metal were performed on 55 × 10 × 2.5 mm sub-size samples of the welded metals at 0 °C [18]. The Vickers microhardness tests of the welded metals were performed on the metallographic samples along a 2 mm vertical line away from the weldments’ surface, as depicted in Figure 4, with a 500 g load and a 10 s dwell time.

## 3. Results and Discussion

### 3.1. Microstructure

#### 3.1.1. Phase Balance and Grain Size

**Figure 5:**
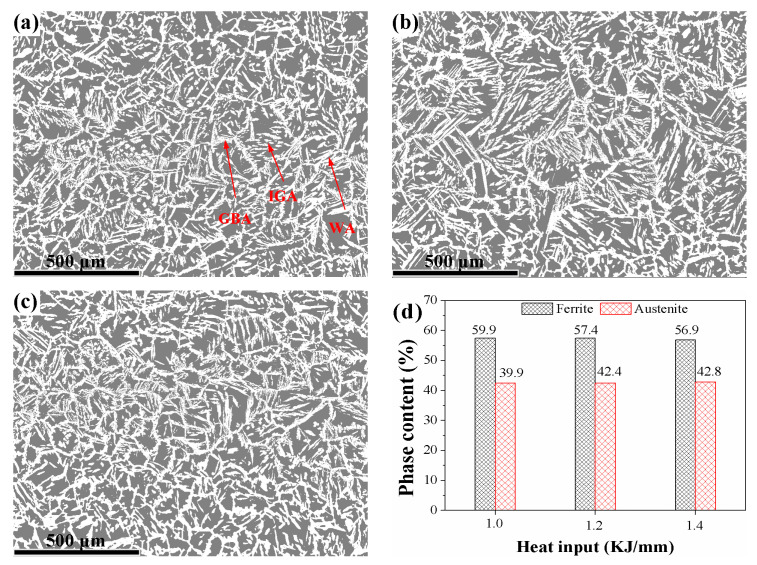
Phase maps of the welded metals with three heat inputs: (**a**) 1.0 kJ/mm, (**b**) 1.2 kJ/mm, (**c**) 1.4 kJ/mm, and (**d**) ferrite and austenite contents of the welded metals. (In (**a**–**c**), the white and grey colors represent austenite and ferrite, respectively.)

Figure 5 is a micrograph of the weld metal calculated by EBSD for three different heat input conditions and the ferrite and austenite contents. The results of the calculation of the austenite and ferrite contents in Channel 5 HKL utilizing the EBSD “phase” component are displayed in Figure 5d. The white-colored austenite showed the characteristic morphologies of Widmanstätten-like austenite (WA), grain boundary austenite (GBA), and intragranular austenite (IGA); and it was discovered dispersed throughout the grey ferrite matrix. The formation of GBA, WA, and IGA depends on the under cooling of the molten pool, respectively, in the range of 800–1350 °C, 650–800 °C, and a lower temperature [8]. When the heat input was enhanced from 1.0 to 1.2 kJ/mm, the austenite ratio went up from 39.9% to 42.4%. Despite an increase in heat input from 1.2 to 1.4 kJ/mm, there was no change in austenite content (between 42.4% and 42.8%). A well-balanced ferrite–austenite microstructure can prevent the formation of detrimental precipitations such as chromium nitrides and σ, leading to increased tensile strength and impact resistance. Since austenite is a softer phase than ferrite, higher concentrations of it result in greater plasticity and improved toughness [19]. There were no welded metal deposits discovered in this investigation. According to Lippold and Kotecki, all weld metals have a 30% minimum phase content because neither ferrite nor austenite contains harmful phases [20].

**Figure 6:**
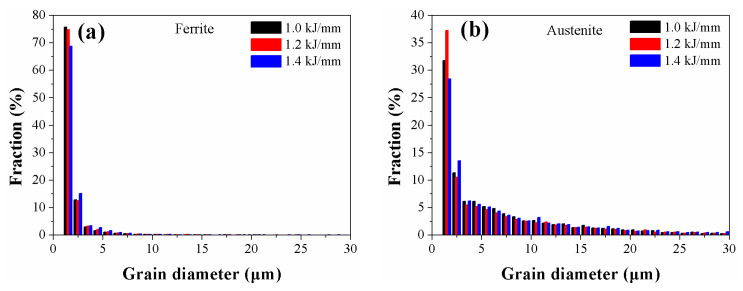
Distributions of austenite and ferrite grain sizes in welded metals with varying HI values (**a**) ferrite and (**b**) austenite.

Figure 6 shows the change in grain size of austenite and ferrite in the welded metals. Table 3 shows the detailed statistics of austenite and ferrite grain sizes. According to Figure 6, austenite and ferrite have diameters that are primarily distributed between 0 and 5, and 0 and 10 μm, respectively. Ferrite had mean grain sizes of 3.60 μm, 3.50 μm, and 4.30 μm, respectively, with 1.0, 1.2, and 1.4 kJ/mm, while austenite had mean grain sizes of 7.44 μm, 7.43 μm, and 8.11 μm, respectively. Typically, a high heat input results in coarse grains during welding. This study found that the size of the ferrite grains did not increase, but rather, slightly decreased as the heat input was raised from 1.0 to 1.2 kJ/mm. This may be because the austenite content increased, which may have limited the growth of the ferrite grains to some extent since austenite nucleated at the ferrite grain boundaries. The ferrite and austenite grains had more time to continue growing when the heat input reached 1.4 kJ/mm, resulting in the largest ferrite and austenite grain sizes.

#### 3.1.2. Interphase Boundary and Σ3 Grain Boundary

The phase interface, grain boundary, and other elements have a significant impact on the material’s mechanical properties in dual-phase alloys. In the duplex stainless steel welding process, the adjacent austenite and ferrite’s crystallographic orientations must follow a coherent or semicoherent orientation relationship (OR), primarily with Nishiyama–Wassermann (N–W) or Kurdjumov–Sachs (K–S) ORs as the crystallographic state. Both ORs were only 5.26° off the parallel close-packed plane normal of the two phases; however, the actual ORs in the ferrite–austenite phase interphase commonly strayed greatly from the ideal K-S/N-W ORs. Ferrite–austenite phase borders that varied no more than 6° from the ideal K-S OR were classified as exceptional interphase boundaries, whereas other interphase boundaries were classified at random [21]. The ideal K–S OR was composed of <110> austenite//<111> ferrite, and {111} austenite//{110} ferrite. It is well-known that the special ferrite–austenite phase interface can make it easier for cracks to spread to the ferrite while, at the same time, stress concentration is created in the particular phase due to the difference between ferrite and austenite’s deformation characteristics [22]. Duplex stainless steel’s grain boundaries were studied, and it was discovered that the low-energy coincidence site lattice (CSL) grain boundaries, specifically the Σ3 twin grain boundaries connected to a 60° misorientation parallel to axis <111> of crystallography, help optimize the properties of the interface, leading to increased crack resistance and, consequently, ductility and toughness [23]. Following Zhang et al., Σ3 grain boundaries with low energy and the random phase interface in austenite are significantly correlated with the impact resistance of the welded duplex stainless steel materials [24].

**Figure 7:**
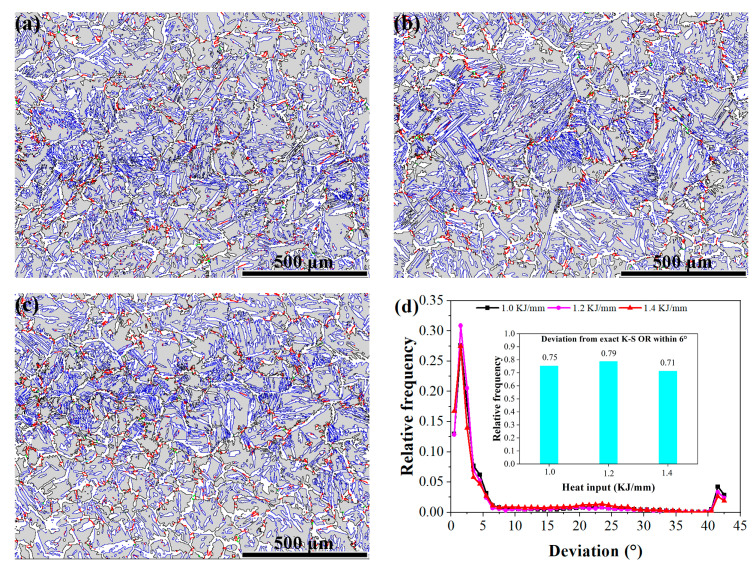
Σ3 twin grain boundaries and interphase boundaries of (**a**) 1.0 kJ/mm, (**b**) 1.2 kJ/mm, (**c**) 1.4 kJ/mm, and (**d**) interphase boundary distribution curve related to deviation in the angular direction from the precise K–S OR boundaries; and the relative proportion of unique interphase boundaries in welded metals with various heat inputs. (Note: in (**a**–**c**), the colors green, black, and red correspond to special interphase boundaries, random interphase boundaries, and Σ3 twin grain boundaries, respectively.)

The relative percentages of the weld metal’s unique phase interface at 1.0, 1.2, and 1.4 kJ/mm are shown in Figure 7, together with the phase interface distribution in relation to angular deviations from the accurate K–S OR. The interphase boundaries of these systems deviated by 0.5 to 42.5° from the precise K–S OR, as illustrated in Figure 7, and the majority of the interface boundaries are special ORs. Almost all WAs, IGAs, and GBAs presented special ORs with adjacent ferrite. According to Figure 7d, the number of unique interphase boundaries that exist in the 1.0 kJ/mm weld metal was greater than in the 1.4 kJ/mm, but lower than in the 1.2 kJ/mm. In the 1.4 kJ/mm welding metal, the content of the special interphase boundaries is very small, indicating that the crack resistance of the sample is stronger. According to Haghdadi et al., increasing the cooling rate leads to more distinctive ferrite–austenite interphase boundaries [25]. The total number of distinct interphase boundaries in the weld metals changes as a function of the welding process’s heat inputs.

Among the 1.0, 1.2, and 1.4 kJ/mm weld metals, the Σ3 austenite twin grain boundaries accounted for 15.5%, 15.9%, and 21.4%, respectively. Crack resistance, toughness, and plasticity are all enhanced by a weld metal’s high proportion of Σ3 grain boundaries; and the toughest weld metal had a plasticity and toughness of 1.4 kJ/mm, followed by 1.2 and 1.0 kJ/mm. Furthermore, when 1.0 to 1.4 kJ/mm of heat is added, the percentage of austenite grain boundaries with Σ3 twins changes similarly to the austenite content, as demonstrated in Figure 5 and Figure 7.

#### 3.1.3. Grain Types

Welding is a complicated process involving synchronized thermal cycles and different types of stresses, including phase transformation and thermal stresses. The residual stress will cause local grain deformation of the austenite and ferrite if the resulting residual stress is greater than the yield strength of the duplex stainless steel material. Plastic deformation, as reported by Haghdadi et al., can lead to the formation of deformed and substructured microstructures [26]. The partially deformed grains of austenite and ferrite spontaneously recrystallize during the subsequent cooling process. Recrystallization is the mechanism by which ferrite and austenite soften [27]. According to this viewpoint, the mechanical characteristics of duplex stainless steel welding materials are influenced by ferrite and austenite grains, particularly the impact of recrystallization.

**Figure 8:**
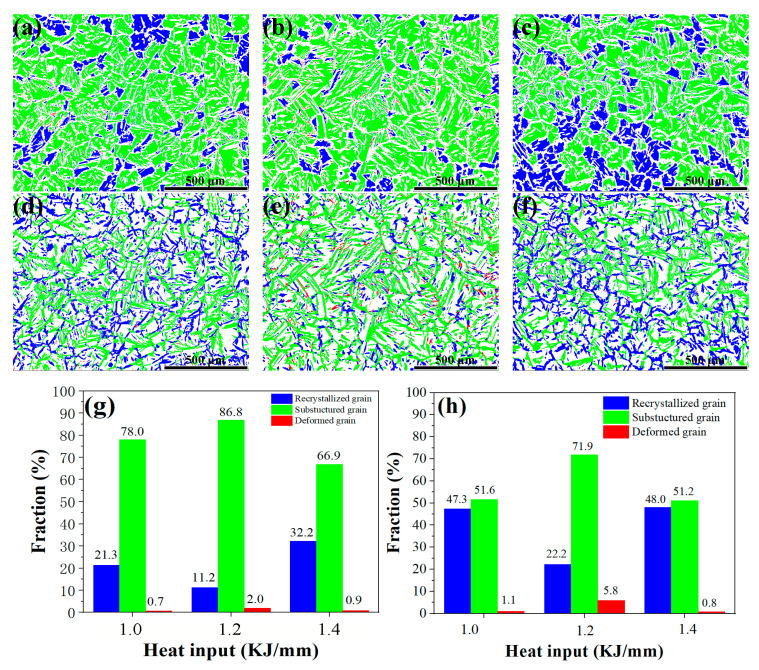
Ferrite and austenite with different grain types obtained with different heat inputs. Maps of the recrystallization calculation for ferrite (**a**–**c**) and austenite (**d**–**f**) with heat inputs of (**a**,**d**) 1.0 kJ/mm, (**b**,**e**) 1.2 kJ/mm, and (**c**,**f**) 1.4 kJ/mm. Histograms of the proportion of various ferrite (**g**) and austenite (**h**) grain types (green for substructured grains, blue for recrystallized grains, and red for deformed grains in (**a**–**f**)).

The relative frequency and distribution of various grain types of austenite and ferrite (such as substructured grains, recrystallized grains, and deformed grains) in the weld metal are depicted in Figure 8. According to the results, the majority of austenite and ferrite grains have a green substructure. In contrast, each weld metal contained a few deformed grains in the austenite and ferrite. In general, there were more recrystallized grains of austenite and ferrite in the weld metals at 1.0 and 1.4 kJ/mm than at 1.2 kJ/mm. Particularly in the 1.4 kJ/mm weld metal, the recrystallized ferrite grains make up 48.0%, which is considerably greater than the other recrystallized austenite and ferrite grains. When there is a greater frequency of formation of recrystallized grain, a material’s plasticity and ductility are significantly improved.

### 3.2. Mechanical Performances

#### 3.2.1. Tensile Test

**Figure 9:**
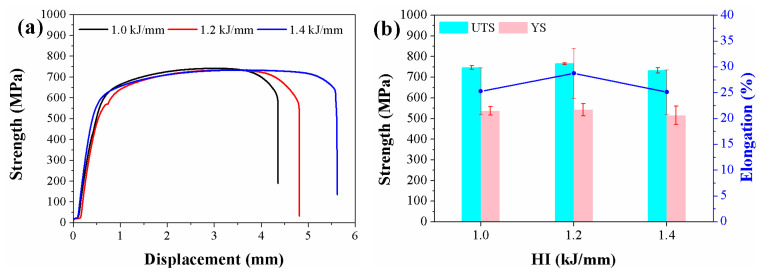
Tensile tests of the welded metals (**a**) tensile curves and (**b**) the UTS, YS and elongation values.

In Figure 9 and Table 4, the tensile results for the three samples at various heat inputs are displayed. From the tensile results obtained, the ultimate tensile strength (UTS) increased first from 746 MPa to 763 MPa, then decreased to 733 MPa with increasing heat input. When the heat input was raised from 1.0 to 1.2 kJ/mm, the yield strength (YS) increased from 538 MPa to 542 MPa, but fell to 515 MPa when it was raised to 1.4 kJ/mm. When the heat input was 1.2 kJ/mm, the elongation reached the maximum value of 28.8%, while the elongation of welded metal with heat inputs of 1.0 and 1.4 kJ/mm differed little, at 25.3% and 25.1%, respectively.

**Figure 10:**
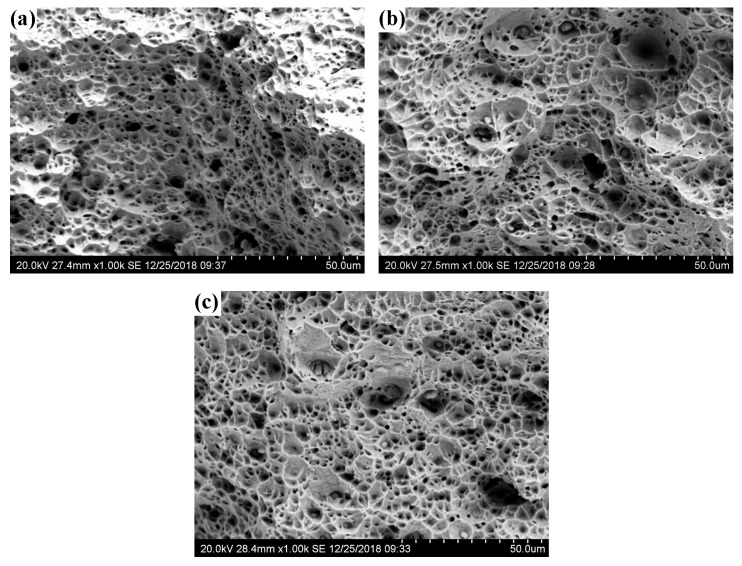
SEM fractographs following tensile tests on the welded metals of various HI: (**a**) 1.0 kJ/mm; (**b**) 1.2 kJ/mm; and (**c**) 1.4 kJ/mm.

The welded metal tensile samples’ microscopic fracture surfaces are displayed in Figure 10. The majority of the fracture mechanisms of the three welded metals were ductile. On the fracture surfaces, dimples of varying sizes were observed surrounding the inclusions. The size of fracture dimples of the three samples had little difference, which may be related to the small difference in austenite grain size shown in Figure 6 and Table 3.

#### 3.2.2. Impact Toughness

**Figure 11:**
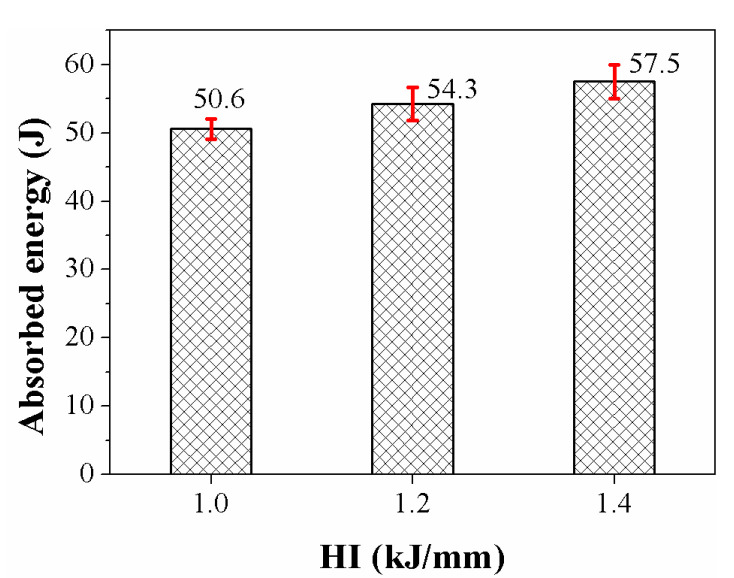
Absorbed energy of the welded metals.

**Figure 12:**
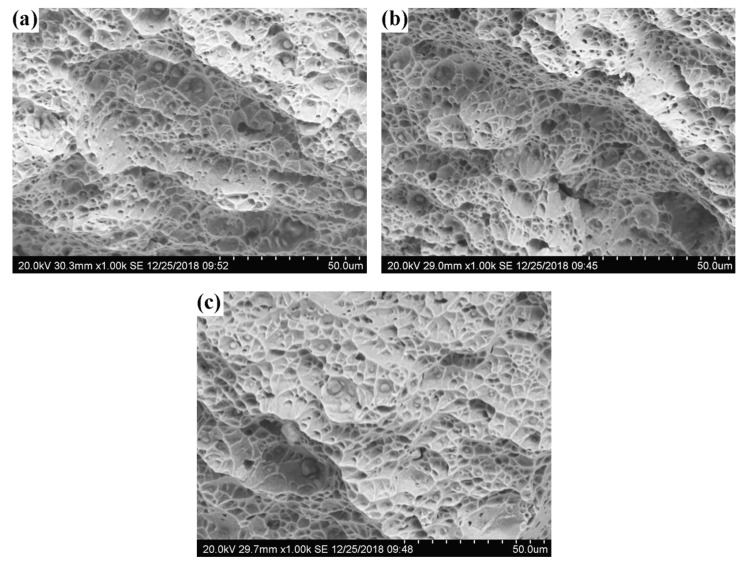
Microfractographs following impact tests on welded metals of various HI: (**a**) 1.0 kJ/mm; (**b**) 1.2 kJ/mm; and (**c**) 1.4 kJ/mm.

In terms of strength and plasticity, a material’s toughness refers to its capacity to absorb energy during fracture and plastic deformation. Charpy V-notch impact tests were carried out at 0 °C to evaluate the impact resistance of the welded metals. As illustrated in Figure 11, all of the specimens’ absorbed energy increased with increasing the heat input. At 1.0 kJ/mm, the average absorbed energy of the welded metal was 50.6 J, which is less than the average value of 54.3 J at 1.2 kJ/mm. The welded metal’s average energy absorption increased to 57.5 J at 1.4 kJ/mm. The fractured surface morphologies following the impact tests are displayed in Figure 12.

Figure 12 shows that at 1.0 and 1.2 kJ/mm, the fractured surfaces of the welded metals were flatter than they were at 1.4 kJ/mm. All welded metals showed numerous dimples and inclusions of various sizes, proving that ductile fracture was the cause of the impact tests’ fracture mechanism. However, compared to the welded metals at 1.0 and 1.2 kJ/mm, the dimples in the welded metals at 1.4 kJ/mm had a greater degree of deformation and a larger average size. Additionally, a few microholes that were caused by inclusions peeling off as a result of deformation from impact testing were visible in the images. Higher impact toughness and elongation in duplex stainless steel samples are typically associated with a higher austenite content [19].

#### 3.2.3. Microhardness

**Figure 13:**
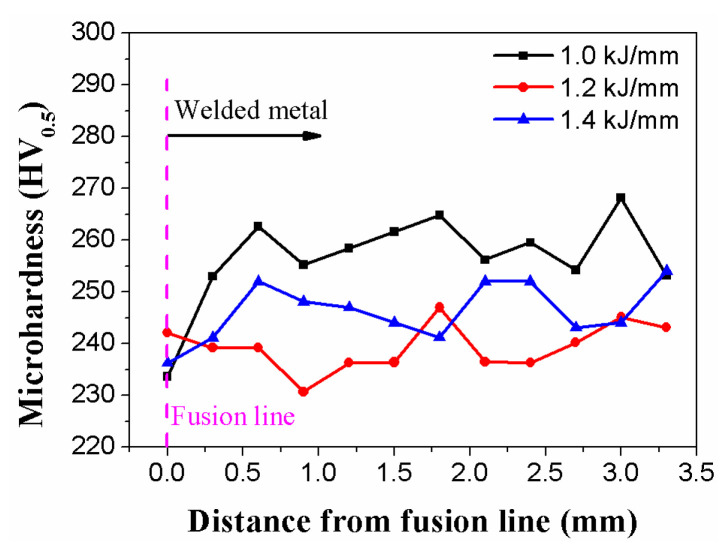
Microhardnesses across half of the welded metals.

The microhardness of the welded section was assessed 2 mm beneath the surface. The distribution of microhardness for the welded samples with heat inputs of 1.0, 1.2, and 1.4 kJ/mm is presented in Figure 13. As illustrated in Figure 13, the heat input changed the weld metal’s microhardness. With a mean value of 238.9 HV_0.5_, it is evident that the weld metal had the lowest microhardness at 1.2 kJ/mm. The two other weld metals had an average microhardness of 258.8 HV_0.5_ (1.0 kJ/mm) and 247.1 HV_0.5_ (1.4 kJ/mm), respectively. The results show that the average microhardness of the welded material changes in a V-shaped curve under different heat inputs.

## 4. Conclusions

Underwater duplex stainless steel welded joints were obtained by a local dry welding method with various heat inputs, and the microstructures and mechanical properties of the welded metals were studied. The results can be concluded as follows:

(1) The amount of austenite and Σ3 Twin austenite grain boundaries’ fractions in the weld metals increased in proportion to the heat input. Austenite and ferrite’s average grain sizes first became fine, and then became significantly larger with increasing welding energy input. The K–S special interphase boundaries showed a trend of increasing first and then decreasing with increasing heat input, which was like the change rule of the substructure grains and deformed grain ratio, but was opposite to the change rule of the recrystallized grain ratio.
(2) As the heat input was increased, UTS, YS, and elongation all initially increased, but then decreased, whereas the amount of energy that the weld metal absorbed increased. The microhardness first decreased and then increased. SEM fractographs of the tensile and impact testes all showed ductile fracture with numerous dimples and inclusions of various sizes.
(3) Higher change values were observed for plasticity and toughness of weld metal when the heat input was raised from 1.0 to 1.2 kJ/mm, compared to those observed when the heat input was increased from 1.2 to 1.4 kJ/mm. Considering energy conservation, it is recommended to adopt 1.2 for welding heat input for better plasticity and toughness.
(4) The results of this article enrich the basic research of underwater welding technology of duplex stainless steel, and can provide certain experimental and theoretical support for the underwater welding of duplex stainless steel. However, this study was conducted under laboratory conditions, without considering other interference factors that may exist in the actual underwater welding process, such as water flow speed, impurities, water salinity, and PH value. The research results are only for reference in practical engineering applications.
(5) This research can be extended in the following aspects: study the thermal cycles in the welding process with different heat input under the condition of greater water depth, or study the corrosion resistance of joints.

## Figures and Tables

**Table 1:** Chemical compositions of UNS S32101 and Supercore 2205P (wt. %).

Materials	C	Si	Mn	Cr	Ni	Mo	Cu	Co	P	S	N	Fe
UNS S32101	0.017	0.49	4.98	21.52	1.56	0.22	0.16	0.04	0.02	<0.002	0.24	Bal.
Supercore 2205P	0.024	0.63	1.57	22.07	8.17	3.05	0.019	-	0.021	0.009	0.15	Bal.

**Table 2:** Welding parameters utilized for underwater one-pass local dry welds.

Specimens	Welding Current (A)	Arc Voltage (V)	Welding Speed (mm/s)	Heat Input (kJ/mm)
#1	200	30	5.6	1.0
#2	200	30	4.8	1.2
#3	200	30	4	1.4

**Table 3:** Statistics on the grain sizes of austenite and ferrite.

	Heat Input (kJ/mm)	Number of Grains	Maximum Value (um)	Minimum Value (um)	Average Value (um)	Standard Deviation (um)	Coefficient of Variation
Ferrite	1.0	7944	196.43	1.6926	3.5937	10.155	2.8258
	1.2	6501	288.21	1.6926	3.4905	10.254	2.9377
	1.4	6438	156.97	1.6926	4.2958	10.846	2.5248
Austenite	1.0	5663	87.965	1.6926	7.4398	8.4074	1.1301
	1.2	5361	97.319	1.6926	7.4290	9.1912	1.2372
	1.4	5046	77.007	1.6926	8.1101	9.1744	1.1312

**Table 4:** Average results of the mechanical tests on the welded metals.

	1.0 kJ/mm	1.2 kJ/mm	1.4 kJ/mm
UTS (MPa)	746 ± 8.3	763 ± 3.6	733 ± 7.7
YS (MPa)	538 ± 8.5	542 ± 12.7	515 ± 7.6
Elongation	25.3 ± 4.53	28.8 ± 4.88	25.1 ± 4.28

## Data Availability

Data sharing not applicable; all the data created for this study are already displayed in the article.
